# Deletion of quinolinate phosphoribosyltransferase gene accelerates frailty phenotypes and neuromuscular decline with aging in a sex‐specific pattern

**DOI:** 10.1111/acel.13849

**Published:** 2023-04-20

**Authors:** Tae Chung, Taylor Bopp, Chris Ward, Francesca M. Notarangelo, Robert Schwarcz, Reyhan Westbrook, Qian‐Li Xue, Jeremy Walston, Ahmet Hoke

**Affiliations:** ^1^ Department of Physical Medicine and Rehabilitation Johns Hopkins University School of Medicine Baltimore Maryland USA; ^2^ Department of Neurology Neuromuscular Division Johns Hopkins University School of Medicine Baltimore Maryland USA; ^3^ Department of Orthopedics University of Maryland School of Medicine Baltimore Maryland USA; ^4^ Maryland Psychiatric Research Center Department of Psychiatry University of Maryland School of Medicine Baltimore Maryland USA; ^5^ Department of Geriatric Medicine and Gerontology Johns Hopkins University School of Medicine Baltimore Maryland USA

**Keywords:** age‐associated muscle weakness, aging, frailty, kynurenine, neurodegeneration, neuromuscular junction, quinolinic acid, sarcopenia

## Abstract

Decline in neuromuscular function with aging is known to be a major determinant of disability and all‐cause mortality in late life. Despite the importance of the problem, the neurobiology of age‐associated muscle weakness is poorly understood. In a previous report, we performed untargeted metabolomics on frail older adults and discovered prominent alteration in the kynurenine pathway, the major route of dietary tryptophan degradation that produces neurotoxic intermediate metabolites. We also showed that neurotoxic kynurenine pathway metabolites are correlated with increased frailty score. For the present study, we sought to further examine the neurobiology of these neurotoxic intermediates by utilizing a mouse model that has a deletion of the quinolinate phosphoribosyltransferase (QPRT) gene, a rate‐limiting step of the kynurenine pathway. QPRT^−/−^ mice have elevated neurotoxic quinolinic acid level in the nervous system throughout their lifespan. We found that QPRT^−/−^ mice have accelerated declines in neuromuscular function in an age‐ and sex‐specific manner compared to control strains. In addition, the QPRT^−/−^ mice show premature signs of frailty and body composition changes that are typical for metabolic syndrome. Our findings suggest that the kynurenine pathway may play an important role in frailty and age‐associated muscle weakness.

## Graphical abstract

The kynurenine pathway is a major route of dietary tryptophan degradation that produces neurotoxic intermediate metabolites. In the present study, we utilized a mouse model that has elevated neurotoxic quinolinic acid level throughout their lifespan. We found that these mice have accelerated declines in neuromuscular function and premature signs of frailty, in an age‐ and sex‐specific manner compared to control strains. Our findings suggest that the kynurenine pathway is associated with increased frailty and age‐associated muscle weakness. Image created with BioRender.com.

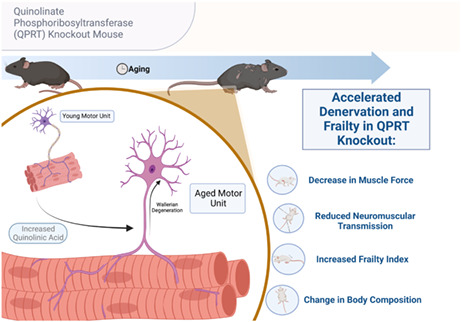

## 1 INTRODUCTION

Decline in neuromuscular function with aging is known to be a major determinant of disability and all‐cause mortality in late life (Ferrucci et al., 2012; Gonzalez‐Freire et al., 2014; Guralnik et al., 1994, 1995; Newman et al., 2006; Sabia et al., 2014; Studenski et al., 2011; Vazzana et al., 2010). Despite the importance of the problem, the neurobiology of age‐associated muscle atrophy and weakness, termed sarcopenia, is poorly understood. To date, various molecular mechanisms of sarcopenia have been proposed, including muscle stem cell dysfunction (Daussin et al., 2021), inflammaging (Daussin et al., 2021; Kalinkovich & Livshits, 2016), mitochondrial dysfunction (Daussin et al., 2021; Ferri et al., 2020), and protein degradation (Tan et al., 2020), among others. However, interaction between metabolic environment, aging, and the neuromuscular system has not been widely studied in sarcopenia and frailty research. Considering that neurons and muscle cells are post‐mitotic and nondividing, systemic changes in metabolism throughout the lifespan likely impact the neuromuscular system. Therefore, changes in the specific metabolic pathways that drive systemic aging may also be responsible for age‐associated decline in neuromuscular function.

In our recent study, metabolomic profiling of frail older adults and a mouse model of frailty showed significant alteration of the kynurenine pathway (Westbrook et al., 2020). This metabolic cascade is a major route of dietary tryptophan degradation and produces neurotoxic intermediate metabolites, which have been suggested to play a role in various neurodegenerative diseases of central nervous system (CNS; e.g., Parkinson's disease, Huntington's disease, and Alzheimer's disease; Schwarcz & Stone, 2017). We showed previously that neurotoxic kynurenine pathway metabolites that are elevated in the serum of frail subjects, namely 3‐hydroxykynurenine (3HK) and quinolinic acid (QA), cause neurotoxicity to peripheral motor neurons in vitro, similar to their neurotoxicity to CNS neurons (Chiarugi et al., 2001; Guidetti & Schwarcz, 1999; Guillemin et al., 2001; Westbrook et al., 2020). The kynurenine/tryptophan ratio was also strongly correlated with serum levels of inflammatory cytokines, including IL‐6 and tumor necrosis factor‐α receptor one (TNF‐ αR1), and with frailty and slow walking speed. These findings paralleled a growing body of evidence suggesting that kynurenine metabolites may induce cytotoxicity in a variety of tissues, including endothelial (Mangge et al., 2014; Wang et al., 2014), renal (Addi et al., 2018; Hsu & Tain, 2020), and retinal (Rejdak et al., 2011) cells. Together, this body of evidence suggests that age‐ and inflammation‐related shunting of tryptophan degradation toward the kynurenine pathway plays a critical role in sarcopenia, frailty phenotypes, and, more generally, physical deterioration late in life.

To further investigate how the neurotoxic metabolite QA may be involved in age‐associated decline in neuromuscular function and frailty, we used a mouse model with a genetic deletion of quinolinate phosphoribosyltransferase (QPRT), a rate‐limiting enzyme of the kynurenine pathway (Gholson et al., 1964). QPRT knockout mice have elevated QA levels in the nervous system throughout their lifespan (Fukuoka et al., 2012). The present study was designed to examine the effects of these hyperphysiological QA levels in the context of the aging process by comparing neuromuscular functions and other phenotypes in QPRT^−/−^ mice and background‐matched wild type mice of both sexes over time.

## 2 RESULTS

### 2.1 Quinolinic acid (QA) is elevated in the spinal cords of QPRT^−/−^ mice

**FIGURE 1:**
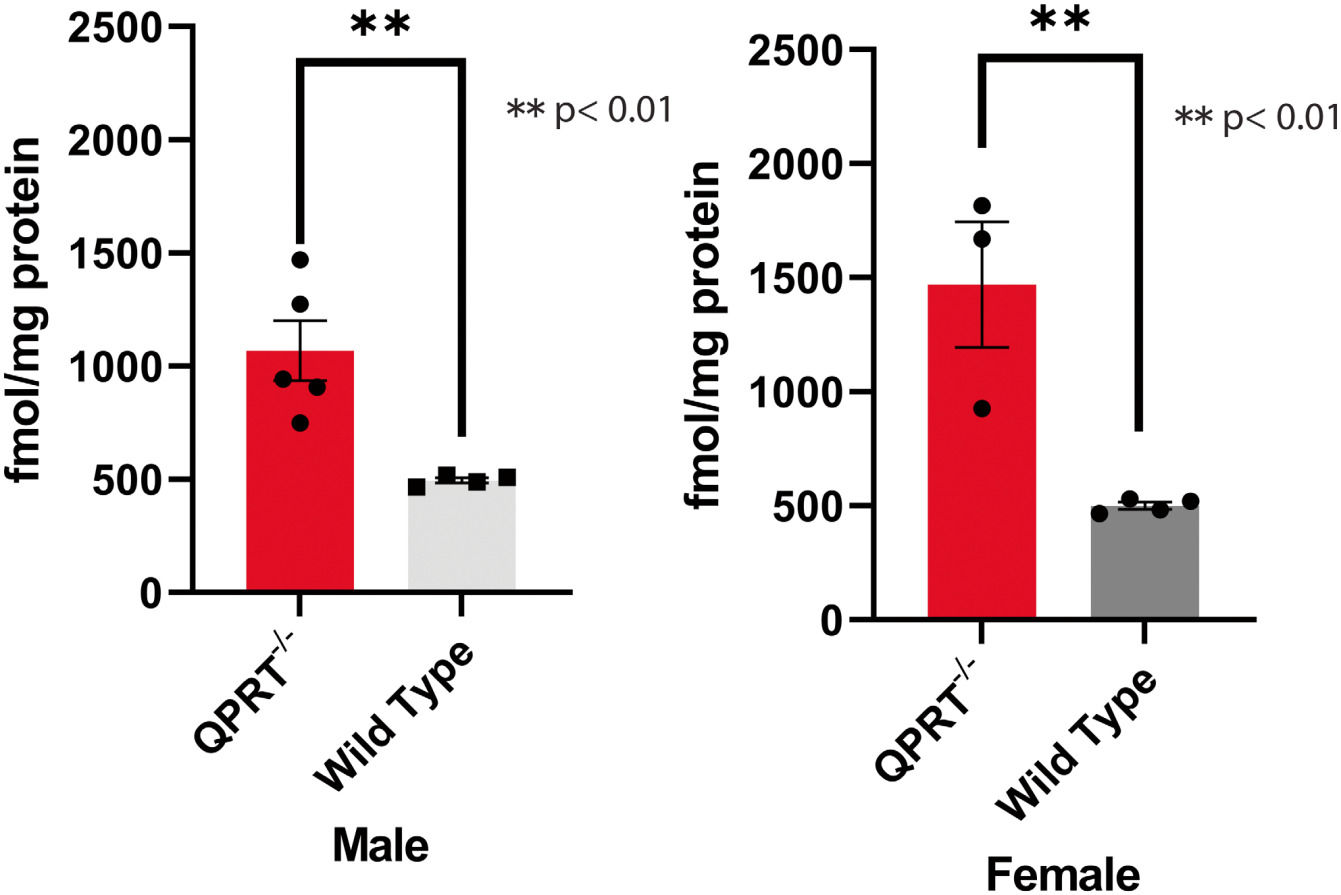
Level of QA in spinal cords of QPRT^−/−^ mice and wild type mice. QA level of female QPRT^−/−^ (*n* = 3) is almost 3‐fold higher than that of female wild type (*n* = 4). QA level of male QPRT^−/−^ (*n* = 5) is also about 3‐fold higher than that of male wild type (*n* = 4). Of the three female QPRT^−/−^ mice, two were 17‐month‐old, one was 6 months old. Of the five male QPRT^−/−^ mice, two were 17‐month‐old and 3 were 6 months old. All eight of the wild type mice were 6 months old. The QA levels of the two 17‐month‐old female mice were 1668.90 and 1815.92 fmol/mg protein, whereas that of young female QPRT^−/−^ mice (6‐month‐old) was 926 fmol/mg protein. The QA level of the two 17‐month‐old male QPRT^−/−^ mice were 941.93 and 908.03 fmol/mg protein, whereas those of 6‐month‐old QPRT^−/−^ mice ranged from 748.03 to 1470.10 fmol/mg protein.

A previous study has shown increased staining of anti‐QA antibody in the brains of QPRT^−/−^ mice, suggesting accumulation of QA in the brain (Fukuoka et al., 2012). Although we expected that QA is elevated in the entire central nervous system, the level of QA in spinal cord of QPRT^−/−^ mice has not been assessed to date. Given that spinal motor neurons are located in the ventral horn of spinal grey matter, we compared the levels of QA in the spinal cord of QPRT^−/−^ of different ages with appropriate wild type mice. We used total of 7 female mice (4 wild type and 3 QPRT^−/−^ mice) and 9 male mice (4 wild type and 5 QPRT^−/−^ mice). The average QA level of male QPRT^−/−^ mice was 495.4 fmol/mg protein and that of male wild type mice was 1069 fmol/mg protein (*t*‐test, *p* = 0.007). The average QA level of female QPRT^−/−^ mice was 499.7 fmol/mg protein and that of female wild type mice was 1470 fmol/mg protein, which was approximately 3‐fold difference (*t*‐test, *p* = 0.009; Figure 1).

### 2.2 Frailty index is significantly higher in old male QPRT^−/−^ mice, but overall lifespan is not affected

**FIGURE 2:**
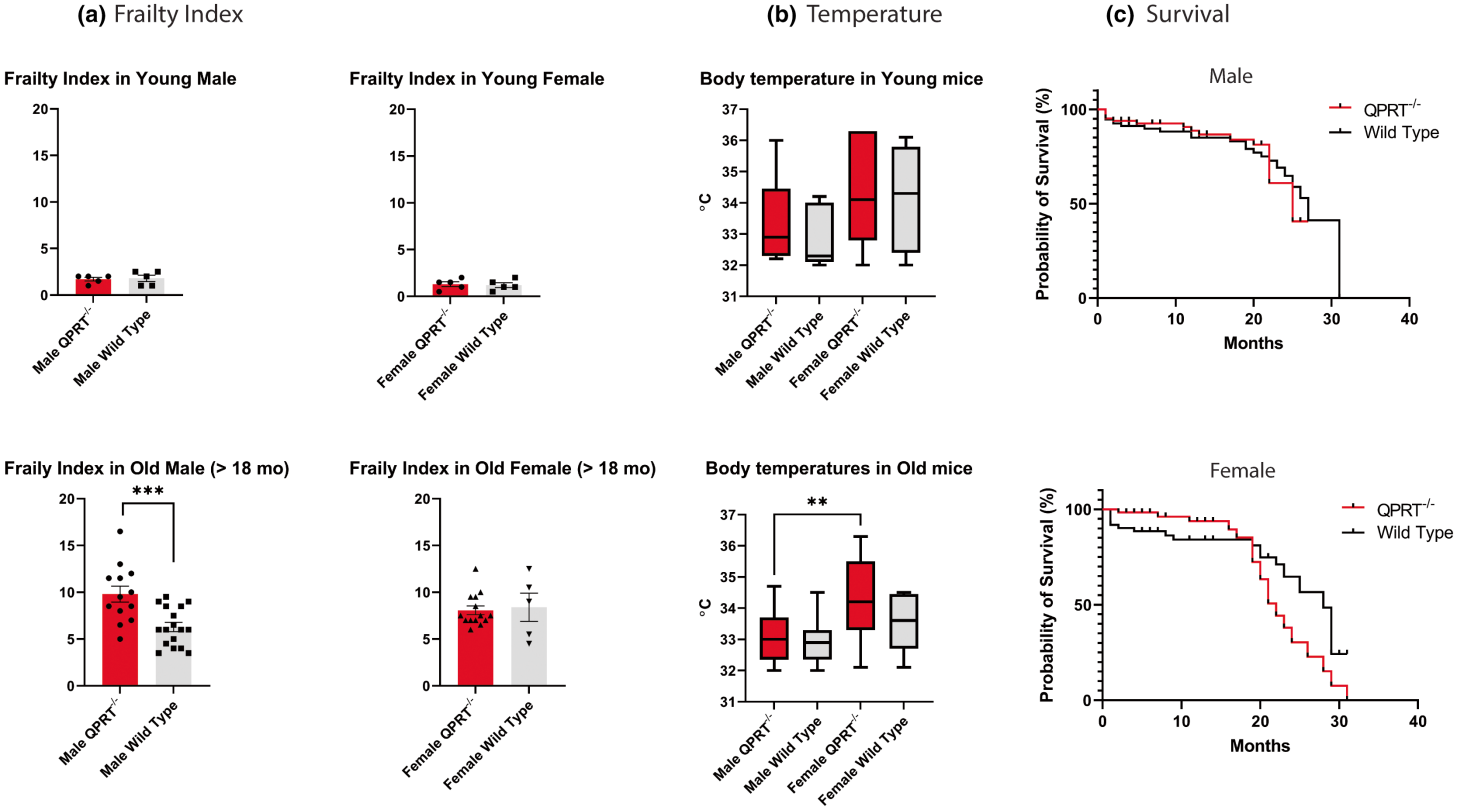
(a) Frailty Index (FI): After 18‐month‐old age, male QPRT^−/−^ mice (*n* = 13) showed higher FI scores as compared to wild type mice (*n* = 17) (*t*‐test, *p* = 0.001). There were no statistically significant differences in FI scores of female mice between the genotypes, although the average FI score of old female QPRT^−/−^ mice (*n* = 14) were higher than those of old female wild type mice (*n* = 5) (*t*‐test, *p* = 0.782). In young mice, there was no difference in FI scores between two genotypes of both sexes (Male QPRT^−/−^ *n* = 5; Male wild type *n* = 5; Female QPRT^−/−^ *n* = 5; Female wild type *n* = 5). (b) Mean body temperatures are generally higher in females than males, although there was no statistical difference across the genotypes and sexes, except for old QPRT^−/−^ mice where mean body temperature of old females is higher than that of old males. (c) Survival Curve: there was no statistically significant difference between the two genotypes in both male and female mice, although there was a trend for lower survivability of QPRT^−/−^ mice.

To assess frailty, we utilized the well‐established Frailty Index (FI) for mice, which measures 31 health‐related variables that provide information about activity levels, hemodynamic status, body composition, and metabolism in mouse models of aging (Whitehead et al., 2014). The total FI score is the sum of scores for each variable, and a high FI predicts adverse outcomes and increased mortality (Howlett & Rockwood, 2013). To examine the effects of age and sex, we used male and female mouse cohort from three age groups: male/female young age (3–6 months), male/female middle age (11–13 months), and male/female old age (>20 months) groups. Comparing the FI scores in QPRT^−/−^ and wild type mice at a young age, we failed to observe differences in FI scores between the two genotypes of either sex (*t*‐test, *p* = 0.806 for male, *p* = 0.789 for female). However, 18‐months‐old male QPRT^−/−^ mice had higher FI scores than age‐matched wild type mice (*t*‐test, *p* < 0.001). Interestingly, although the average FI score of old female QPRT^−/−^ mice was higher than in age‐matched female wild type mice, we did not observe statistically significant genotypic differences (*t*‐test, *p* = 0.781; Figure 2a).

In both young and old mice, body temperature tended to be higher in females than in males, except for old QPRT^−/−^ mice where female mice showed significantly higher body temperature than males (*t*‐test, *p* = 0.009; Figure 2b). There were no sex‐ or genotype‐related statistical differences in the average temperatures between the two age groups. We also compared the life span of QPRT^−/−^ and wild type mice, we noted that the median survival of QPRT^−/−^ mice tended to be shorter than that of wild type mice (male QPRT^−/−^: 25 months; male wild type: 27 months; female QPRT^−/−^: 22 months; female wild type: 28 months), although the differences did not meet statistical significance (Figure 2c).

### 2.3 QPRT^−/−^ mice have altered body composition that is typical for metabolic syndrome

The kynurenine pathway is reportedly altered in metabolic syndrome (Mizutani et al., 2002; Niinisalo et al., 2008; Oxenkrug, 2010), a condition defined by the presence of risk factors specific for cardiovascular diseases, such as abdominal obesity, high blood pressure, impaired fasting blood glucose, and high cholesterol (Pi‐Sunyer, 2019). Notably, increased visceral fat and reduced lean body mass in metabolic syndrome predispose individuals for sarcopenia and decline in neuromuscular functions in late life (Ishii et al., 2014). We hypothesized that increased QA levels in QPRT^−/−^ mice may affect multiple organ systems that contribute to the development of frailty in these animals. We also divided the mouse cohort into three age groups of two sexes as described above to see the effects of age and sex. To evaluate body composition, we compared the weight of whole body, heart, liver, kidney, visceral fat, and skeletal muscle tissues in our experimental animals (Figure S1). Whole body weight was significantly higher in both male and female QPRT^−/−^ mice than in corresponding wild type mice at all ages tested (unpaired *t*‐test, young male *p* < 0.001; young female *p* < 0.001; middle age male *p* < 0.001; middle age female *p* < 0.001; old male *p* = 0.002; old female *p* = 0.002). Moreover, QPRT^−/−^ mice had a larger amount of visceral fat than wild type mice at a young age (unpaired *t*‐test, young male *p* = 0.037; young female *p* = 0.008). While this trend was consistently observed in all age groups and in both sexes, those differences did not reach statistical significance. The weight of liver increased with age in both sexes and genotypes; notably, hepatomegaly was detected in both male and female QPRT^−/−^ mice at a young age, possibly indicating fatty liver considering the increased visceral fats in the same age group (unpaired *t*‐test, young male *p* = 0.026; young female *p* < 0.001). Over time, the difference between the genotypes in the size of the liver diminished with age. The weight of spleen increased in QPRT^−/−^ mice, especially in young males (unpaired *t*‐test, *p* = 0.037). The overall muscle tissue weights were not significantly different between the two genotypes. However, when muscle weights of tibialis anterior were adjusted by total body weights, those of QPRT^−/−^ mice were significantly smaller than wild type at young and middle‐aged mice in both males and females, suggesting that lean body mass is reduced in QPRT^−/−^ mice (unpaired *t*‐test, young male *p* = 0.003; young female *p* = 0.004; middle age male *p* = 0.001; middle age female *p* = 0.013). In old age, muscle weights of tibialis anterior did not differ between the two genotypes.

### 2.4 Old male QPRT^−/−^ mice have reduced grip strength

**FIGURE 3:**
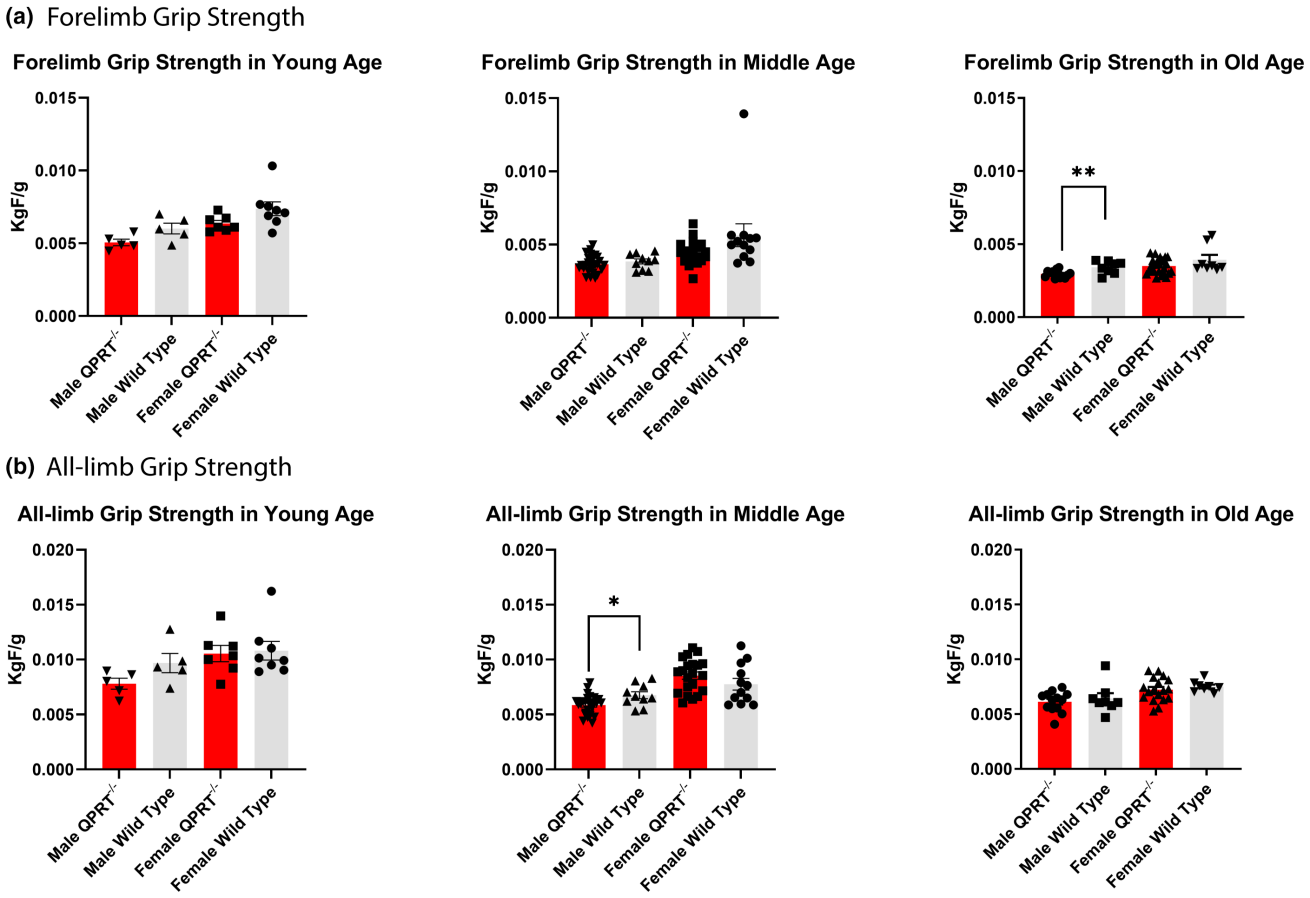
Grip Strength. All grip strength was normalized by total body weight. Overall, male QPRT^−/−^ mice showed reduced grip strength for all‐limbs in both middle (*n* = 26) and old (*n* = 13) age groups. In females, the average grip strength of female QPRT^−/−^ mice was lower than that of wild type, although there was no statistical difference. However, compared to male wild type mice, male QPRT^−/−^ mice showed lower all‐limb grip strength in middle (*p* = 0.014) and forelimb grip strength in old ages (*p* = 0.007).

We measured the grip strength of the mice at all age groups, using a Chatillon force measurement device (Figure 3). We also divided the mouse cohort into three age groups of two sexes as described above to examine the effects of age and sex. As expected, there was no difference in the grip strength across genotypes and sexes in young mice. However, compared to male wild type mice, male QPRT^−/−^ mice showed lower all‐limb grip strength in middle (*t*‐test, *p* = 0.014) and forelimb grip strength in old ages (*t*‐test, *p* = 0.007). In female mice, the average grip strength of female QPRT^−/−^ mice tended to be generally lower than that of wild type animals, although the difference was not statistically significant (old female forelimbs; *t*‐test, *p* = 0.113; old female all‐limbs; *t*‐test, *p* = 0.470). We also used two‐way ANOVA to test the average difference between genotypes, average difference between age groups, and interactions between age and genotypes. In males, there were significant differences in forelimb grip strength by age (*p* < 0.001) and genotypes (*p* = 0.005). In females, there were also significant differences in forelimb grip strength by age (*p* < 0.001) and genotypes (*p* = 0.006). There was no interaction between age and genotypes in forelimb grip strength for both males and females. In addition, in males, there were significant differences in all‐limb grip strength by age (*p* < 0.001) and genotypes (*p* = 0.004). In females, there was a significant difference in all‐limb grip strength by age (*p* < 0.001) but not by genotypes (*p* = 0.603). Moreover, there was no interaction between age and genotypes for all‐limb grip strength. It appears that all‐limb grip strength of female QPRT^−/−^ mice is preserved in middle age, which is a similar pattern of sex difference that was observed in the frailty evaluation where female QPRT^−/−^ mice did not exhibit high FI scores in middle to old age (cf. Figure 1a).

### 2.5 Nerve‐evoked isometric force measurement shows significant reduction of force‐frequent relationship in QPRT^−/−^ mice after middle age

**FIGURE 4:**
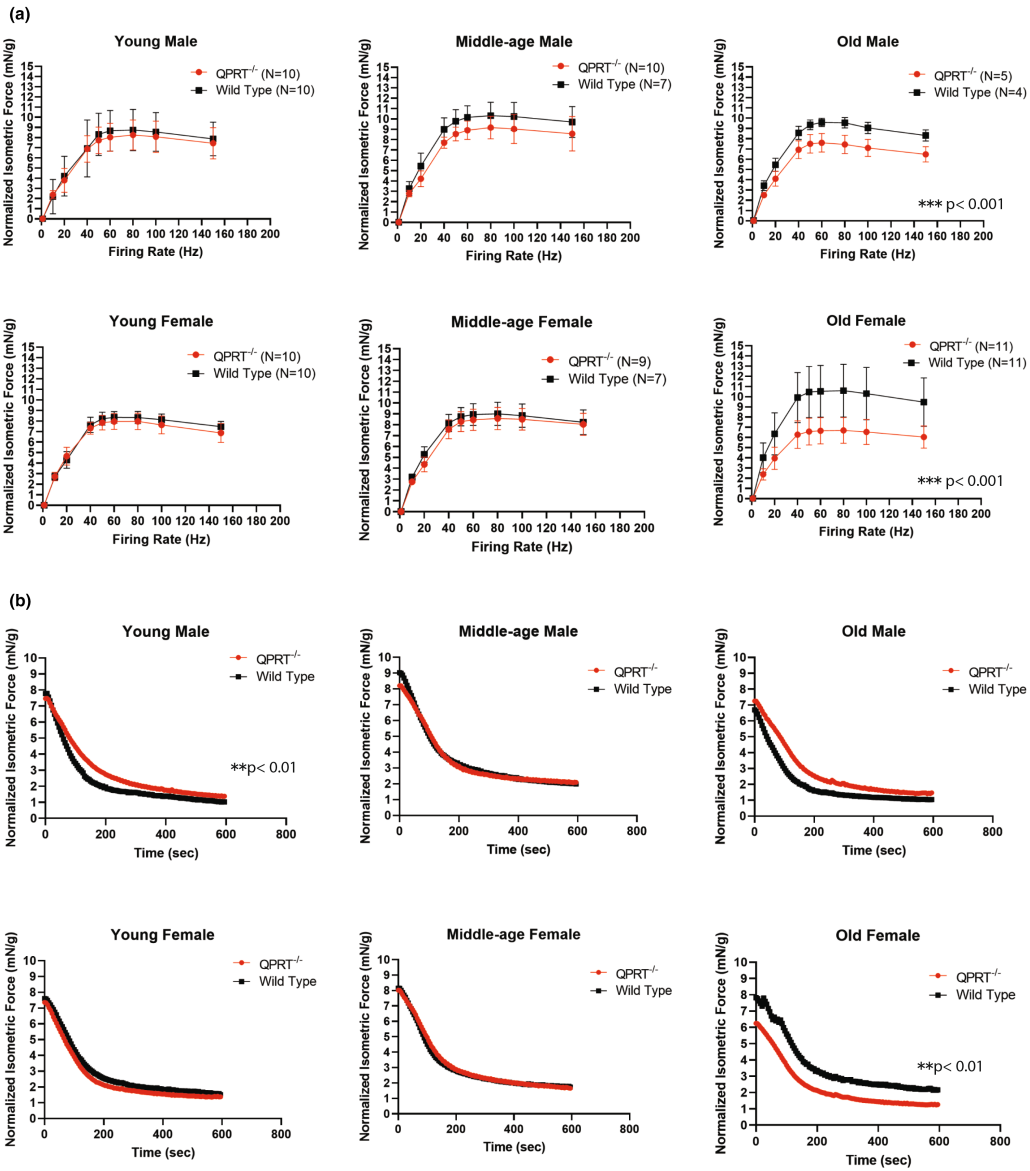
(a) Force‐frequency Curve: In males, maximal isometric force of QPRT^−/−^ mice began to decline in middle age and became significantly reduced by old age. In females, maximal isometric force of QPRT^−/−^ mice was not significantly different until old age, after which that of QPRT^−/−^ mice was dramatically reduced. Repeated measures ANOVA analysis showed significantly reduced curve in QPRT^−/−^ mice as compared to wild type in old age for both sexes). (b) Fatigability: The decline of isometric force over 10 min was observed under tibial nerve stimulation at 80 Hz for 1 s every 5 s. Mixed‐effects regression analyses were used to compare the rate of decline between two genotypes. There was no statistically significant between‐group difference in the fatigability trajectory among young female mice and middle‐aged mice groups of both sexes between the two genotypes. Interestingly, there was significant overall difference between the trajectories (*p* < 0.01), showing reduced rate of decline early on in isometric force of young male QPRT^−/−^ mice as compared to that of young male wild type mice (*p* = 0.06). The same pattern was observed in old age group of both sexes where the rate of initial decline is reduced in QPRT^−/−^ mice as compared to that of wild type mice (*p* < 0.05 for both). In old females, overall fatiguability curve of QPRT^−/−^ mice is significantly reduced as compared to that of wild type mice (*p* < 0.01), but the rate of decline in QPRT^−/−^ mice is significantly reduced as well, suggesting that they are more resistant to fatigue than wild type mice (*p* < 0.01).

Force production is dependent on a variety of factors, including sarcomere length, frequency of motor unit activation, and contractile apparatus (MacDougall et al., 2020). To evaluate muscle contractibility, we measured nerve‐evoked isometric force, normalized by body weight, upon stimulation of tibial nerve to gastrocnemius muscles at frequencies ranging from 1 to 150 Hz (Figure 4a). We compared both maximal isometric force at any given frequency between the two genotypes and genotype‐frequency interaction on the between‐genotype differences over the frequency spectrum. In young males, the average maximal isometric force at any given frequency of QPRT^−/−^ mice was 8.284 mN/g whereas that of wild type mice was 8.798 mN/g. In young females, the average maximal isometric force at any given frequency of QPRT^−/−^ mice was 8.013 mN/g whereas that of wild type mice was 8.385 mN/g. At young age, there was no significant difference in force‐frequency relationship or the maximal isometric force between QPRT^−/−^ and wild type mice. In middle‐aged males, the average maximal isometric force of QPRT^−/−^ mice was 9.263 mN/g whereas that of wild type mice was 10.376 mN/g. Thus, while no statistical significance was observed, QPRT^−/−^ mice tended to have reduced maximal isometric force as compared to wild type mice in this group. In middle‐aged females, the average maximal isometric force at any given frequency of QPRT^−/−^ mice was 8.608 mN/g whereas that of wild type mice was 9.023 mN/g (no significant group difference). In old males, the maximal isometric force at any given frequency of QPRT^−/−^ mice was significantly reduced (average 7.616 mN/g) compared to wild type mice (average 9.619 mN/g, *t*‐test, *p* < 0.001). In old females, too, the maximal isometric force at any given frequency of QPRT^−/−^ mice was significantly reduced (average 6.744 mN/g) compared to wild type controls (average 10.634 mN/g, *t*‐test, *p* < 0.001). As illustrated in Figure 4a, we tested genotype‐frequency interaction on force using repeated measures ANOVA. The slopes of force‐frequency relationship curves remained unchanged until middle age in both male and female mice. Repeated measures ANOVA analysis revealed significant genotype‐frequency interaction in both male and female mice at old age suggesting that the between‐genotype difference in isometric force was greater at higher frequency among old mice s (*F*_8,64_ = 3.97, *p* = 0.04) and females (*F*_8,160_ = 16.12, *p* < 0.001).

To evaluate fatigability, we stimulated tibial nerve at 80 Hz for 1 s every 5 s and evaluated the decline of isometric force over 10 min. The extent of isometric force declines as well as the time to reach the lowest isometric force were compared over 10 min of repetitive stimulation. Mixed‐effects models were used to compare the rate of decline between two genotypes. There was no statistically significant difference in young female mice and middle‐aged groups of mice of both sexes between the two genotypes (Figure 4b). Interestingly, we observed a significant overall difference between the trajectories, showing reduced rate of decline early on in isometric force of young male QPRT^−/−^ mice compared to young male wild type mice (*p* = 0.06). The same pattern was seen in old age groups of both sexes where the rate of initial decline was reduced in QPRT^−/−^ mice compared to wild type mice (*p* < 0.05 for both). However, in old‐age groups, there was a significant reduction in isometric force in female, but not in male, QPRT^−/−^ mice compared to wild type mice (*p* < 0.01). Overall, the sex‐specific patterns of isometric force, force‐frequency curve, and fatigability consistently suggest that overall muscle functions of male QPRT^−/−^ mice decline faster than those of female QPRT^−/−^ mice after middle age.

### 2.6 QPRT^−/−^ mice show reduced compound motor action potential and increased single fiber jitter

**FIGURE 5:**
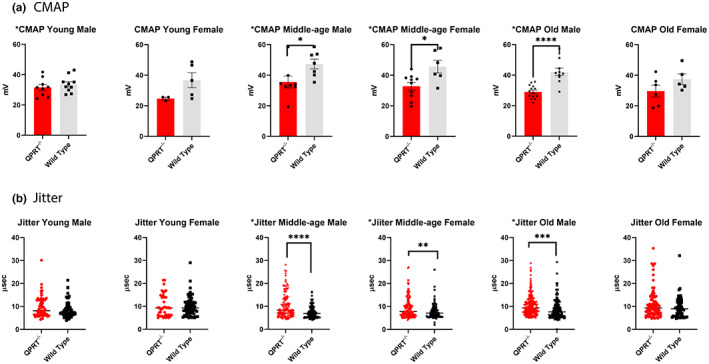
(a) CMAP: There was no statistical difference between the two genotypes in young mice of both sexes. CMAP amplitudes were significantly lower in QPRT^−/−^ mice in middle‐aged male (*p* = 0.038) and middle‐aged female (*p* = 0.014). In old age, only males showed the difference in CMAP amplitudes between the two genotypes (*p* < 0.001), but in old age females, there was no statistical difference between the two genotypes as CMAP amplitudes of wild type female mice declined rapidly after middle age. (b) Single Fiber Jitter: In young age, there was no difference in jitter levels between the two genotypes of both sexes. In middle age, there was significantly higher jitter level in QPRT^−/−^ mice in both males (*p* < 0.001) and females (*p* = 0.009), but the difference was much greater in males. In old age, there was still difference in jitter levels in males (*p* = 0.001), but the statistical difference no longer exists in the females as the jitter level of female wild type mice increases rapidly after middle age.

Failure in neuromuscular transmission has been suggested to be an important contributing factor for sarcopenia with aging (Chugh et al., 2020; Padilla et al., 2021). To further explore neuromuscular transmission and axonal integrity in QPRT^−/−^ mice, we performed nerve conduction study and single fiber electromyography to evaluate how much neuromuscular denervation contributes to the reduced muscle strength of QPRT^−/−^ mice. We showed previously that reduced neuromuscular transmission is associated with reduced motor function with aging, and other studies described neuromuscular denervation in early stages of sarcopenia (Chai et al., 2011; Cheng et al., 2013; Chung et al., 2017, 2018; Doherty et al., 1993; Rowan et al., 2012). To evaluate neuromuscular transmission, we performed single fiber electromyography (SFEMG) and compared jitter levels between the two genotypes (Figure 5). SFEMG jitter represents the variation in time interval between stimulation and single fiber action potentials. High level of jitter in μs sensitively reflects neuromuscular junction defects (Sanders & Stålberg, 1996). At young age, there was no difference in jitter levels between the two genotypes (Mann–Whitney test, male *p* = 0.202, female *p* = 0.723). However, in middle age, jitter levels were significantly higher in QPRT^−/−^ mice in both males (Mann–Whitney test, *p* < 0.001) and females (Mann–Whitney test, *p* = 0.016). In the old‐age group, there was still difference in jitter levels in males (Mann–Whitney test, *p* < 0.001) but was not observed in the females (Mann–Whitney test, *p* = 0.336). To test the average difference between genotypes and age for jitter levels, we also performed two‐way ANOVA. In males, there was a significant difference in jitter by genotypes (*p* < 0.001), but not by age (*p* = 0.221). In females, there were significant differences in jitter by both age (*p* = 0.002) and genotypes (*p* = 0.011). There was no interaction between age and genotypes.

We also compared the amplitudes of compound motor action potential (CMAP) of tibial nerve at gastrocnemius muscle between the two genotypes in both sexes over all age groups (Figure 5). CMAP is the summation of action potentials of the entire stimulated motor nerve. Low CMAP amplitudes primarily suggest reduced motor nerve innervation, although CMAP can be reduced in extreme myofiber atrophy or degeneration. There was no difference in CMAP amplitudes between young QPRT^−/−^ and wild type mice. Consistent with the jitter data (see above), CMAP amplitudes were significantly lower in QPRT^−/−^ mice in middle‐aged males (*t*‐test, *p* = 0.038) and middle‐aged females (*t*‐test, *p* = 0.014). In old age, only males showed the difference in CMAP amplitudes between the two genotypes (*t*‐test, *p* < 0.001). In contrast, no difference between genotypes was noted in old females since CMAP amplitudes of wild type female mice declined rapidly after middle age. When analyzed with two‐way ANOVA, in males, there were significant differences in CMAP by both age (*p* = 0.003) and genotypes (*p* < 0.001), and there was significant interaction between genotype and age such that the difference by age (or genotype) varied by genotype (or age). In females, there was a significant difference in CMAP by genotypes (*p* < 0.001), but not by age (*p* = 0.087). There was no interaction between age and genotype in females. Distal latencies of tibial nerve CMAP showed no differences between the two genotypes in both sexes of all age groups, suggesting that conduction velocity was preserved. Repetitive stimulation was performed at 3 Hz in these mice, and there was no difference in the percentage decrements of CMAP amplitudes between two genotypes in all age/sex groups.

### 2.7 QPRT^−/−^ mice show increased denervation at neuromuscular junction and muscle atrophy after middle age

**FIGURE 6:**
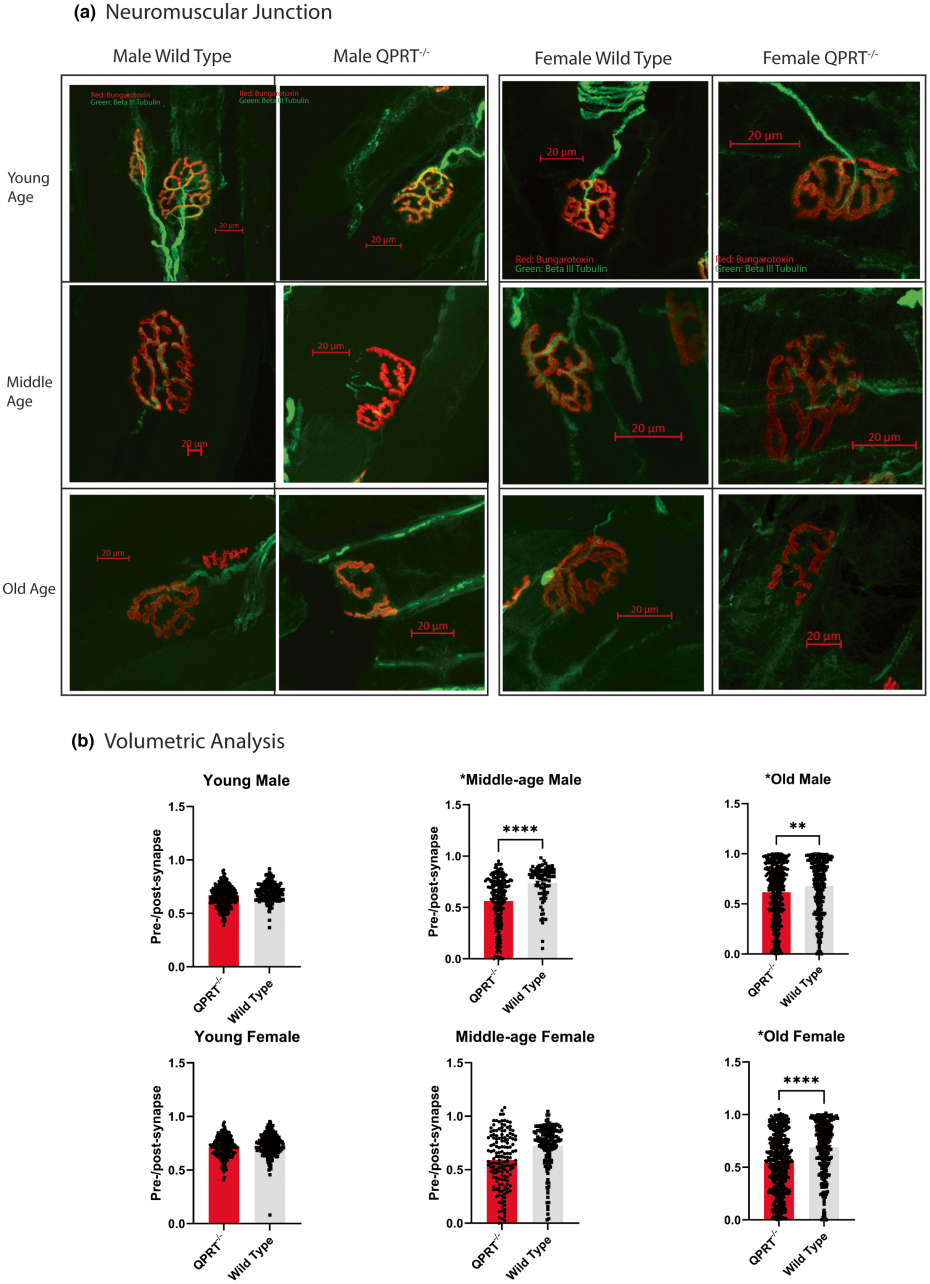
(a) Neuromuscular junction (NMJ) at extensor digitorum longus (EDL) muscles: double immunofluorescent staining was performed on EDL muscles, using beta‐III tubulin (green) as presynaptic marker and bungarotoxin (red) as postsynaptic marker. In young age, there was no difference in the NMJ morphology in both sexes. In middle‐aged mice group, NMJ of male QPRT^−/−^ mice began to show signs of neuromuscular denervation, such as fragmentation and increased branching. In old age, the NMJ of both genotypes show reduced coverage of presynaptic area over postsynaptic area, in addition to increased branching and sprouting. In QPRT^−/−^ mice, there were extensive fragmentations and partial denervation of NMJ on both sexes, starting in middle age. (b) Semi‐quantitative analysis of NMJ Coverage: In young age, there was no difference between NMJ coverage ratio between the genotypes in both sexes. In middle‐aged mice group, NMJ coverage ratio of male QPRT^−/−^ mice (*n* = 5) was significantly reduced as compared to that of wild type (*n* = 11), which was not shown in female mice group (female wild type *n* = 5; female QPRT^−/−^ *n* = 5). In old age, NMJ coverage ratio of both male and female QPRT^−/−^ mice was significantly reduced as compared to that of wild type (male QPRT^−/−^ *n* = 14; male wild type *n* = 8; female QPRT^−/−^ *n* = 13; female wild type *n* = 9).

Given the increased jitter in QPRT^−/−^ mice with aging, we expected that partial denervation at the neuromuscular junction (NMJ) may be a driving event of age‐associated neuromuscular weakness, as also shown in previous studies (Hashizume & Kanda, 1995; McNeil et al., 2005; Tomlinson & Irving, 1977; Wokke et al., 1990). We performed immunofluorescence staining of neuromuscular junctions in soleus muscles, using Bungarotoxin as a presynaptic marker and βIII‐tubulin as a postsynaptic marker. To assess neuromuscular innervation, we compared the ratio of presynaptic coverage over postsynaptic areas in three‐dimensional volumes between QPRT^−/−^ and wild type mice, using Imaris® software on confocal images. In the young‐age group, there was no difference in the NMJ coverage ratio in either sex. In middle age, NMJ of male QPRT^−/−^ mice began to show signs of neuromuscular denervation, such as fragmentation and increased branching (Figure 6a). Semi‐quantitative volumetric analyses of NMJ revealed that the NMJ coverage ratio of middle‐aged male – but not in female – QPRT^−/−^ mice was significantly reduced compared to that of wild type (Mann–Whitney test, *p* = 0.002; Figure 6b). Compared to wild type mice, the NMJ coverage ratio was significantly reduced in both male (Mann–Whitney test, *p* = 0.003) and female (Mann–Whitney test, *p* < 0.001) QPRT^−/−^ mice in old age, that is, partial denervation was greater in old QPRT^−/−^ mice than in old wild type animals (Figure 6b).

Finally, we stained skeletal muscle with hematoxylin and eosin (H&E). H&E staining of cross sections did not reveal differences in endomysial or perivascular inflammation between the two genotypes across all ages. To examine if muscle atrophy may have contributed to the reduced muscle strength of QPRT^−/−^ mice, we compared the cross‐sectional diameters of myofibers in tibialis anterior muscles between the two genotypes (Figure S2). Despite the significant denervation at NMJ, overall cross‐sectional diameters were not different between two genotypes in old male and female mice, suggesting that neurogenic atrophy has not occurred by 20‐months of age in either genotype or sex.

## 3 DISCUSSION

Our study shows that a mouse model with a genetic deletion of QPRT develops muscle weakness and frailty with aging. As QA confers neurotoxicity via the N‐methyl‐D‐aspartate (NMDA) receptor, and as spinal motor neurons are known to express functionally active NMDA receptors throughout the lifespan (Enríquez Denton et al., 2012; Kalb et al., 1992; Manuel et al., 2012; Nagy et al., 2004), spinal motor neurons of QPRT^−/−^ mice may be chronically exposed to toxic levels of QA. We demonstrated that (1) neurotoxic effects of increased QA in the system begin to emerge in middle to old age, and (2) female mice are relatively resistant to QA neurotoxicity until middle age, although their neuromuscular function quickly declines at old age. Interestingly, a similar pattern of sex difference is observed in human frailty in that sarcopenia and frailty are more prevalent and severe in females, as compared to males, in late life (Park & Ko, 2021; Roberts et al., 2018). In both sexes, it is striking that neuromuscular functions are preserved in young‐age group, despite the elevated levels of QA, a potent neurotoxin, in the spinal cord.

There are few possible explanations why young animals are protected from QA neurotoxicity. First, it is possible that chronic exposure of QA over time potentiates neurotoxicity. In fact, as seen in Figure 1, the concentration of QA in the spinal cord of QPRT^−/−^ mice ranges from 465 to 1816 fmol/mg protein. In our previous in vitro experiments, neurotoxic effects of QA were seen at concentrations of 3 to 30 μM, though they were seen at 0.3 μM QA, that is, they were greatly potentiated, when motor neurons were incubated with both QA and its bioprecursor 3‐HK (Westbrook et al., 2020). Therefore, in the spinal cord of QPRT^−/−^ mice, the concentration of QA is roughly 10^5^‐fold lower than the neurotoxic concentration. However, neurotoxicity can occur at submicromolar concentrations of QA after chronic exposure, as shown using rat corticostriatal neurons in vitro (Whetsell & Schwarcz, 1989). Of note in this context, spinal neurons of QPRT^−/−^ mice are exposed to increased concentrations of QA for 12–20 months. Separately or in addition, endogenous protective mechanisms against neurotoxic QA may deteriorate with aging and predispose to QA neurotoxicity in late life (Fukuoka et al., 2012). Interestingly, kynurenine and some downstream metabolites are known to bind and activate aryl hydrocarbon receptor (AhR), which, in turn, suppresses inflammation. Since kynurenines are increased by inflammation through indolamine‐2,3‐dioxygenase (IDO), the kynurenine‐AhR pathway creates a negative feedback loop, preventing continuous production of kynurenine metabolites (Salminen, 2022). Notably, AhR function is known to decline with aging (Bravo‐Ferrer et al., 2019) and may, therefore, predispose older animals to neurotoxicity. Indeed, AhR‐null mice show accelerated brain aging and shortened lifespan (Bravo‐Ferrer et al., 2019).

The pattern of sex differences observed in the present study was consistent throughout the various aspects of neuromuscular functions and frailty phenotype, that is, female mice were shown to be relatively protected from QA toxicity until middle age, though protection became obsolete in old age. This phenomenon may be mechanistically linked to a role of sex hormones. Estrogen increases the activity of the kynurenine pathway (Braidman & Rose, 1971) and, due to the anti‐excitotoxic effects of estrogen receptor activation, prevents the neurotoxic actions of QA (Moursi et al., 1970). Estrogen receptor is neuroprotective against glutamate‐induced neurotoxicity and given that QA is a NMDA receptor agonist, it is possible that the neuroprotection of female QPRT^−/−^ mice is due to reduced excitotoxicity of QA (Lan et al., 2014). In addition, progesterone suppresses the levels of 3‐hydroxyanthranilic acid, anthranilic acid, and kynurenic acid in the post‐ovulatory phase of the menstrual cycle (Moursi et al., 1970). Reduced production of sex hormones in old age may, therefore, explain enhanced neurotoxicity in late life. Additional studies are clearly needed to explore the role of kynurenine pathway metabolites in sex‐specific differences of frailty phenotypes in old age.

While neuromuscular decline and frailty phenotypes are facilitated in QPRT^−/−^ mice, our survival analysis did not reveal significant difference between the two genotypes. Unfortunately, only a small number of mice were available for survival analysis in the present study since animals were sacrificed for tissue harvesting at each age. Notably, however, the median survival of QPRT^−/−^ mice tended to be shorter than that of wild type mice, even though the difference did not meet statistical significance. Given that frailty phenotypes and neuromuscular decline were accelerated in late life, it is possible that survival of QPRT^−/−^ mice is, in fact, significantly shorter than that of wild type as the animals approach 30 months of age.

The striking difference in body composition between QPRT^−/−^ and wild type mice suggests that QPRT^−/−^ mice have increased fat mass and reduced lean body mass that is typical for metabolic syndrome. Sarcopenia is accompanied by systemic changes of the metabolome and by a significant reduction of lean body mass (Korostishevsky et al., 2016; Lu et al., 2020; Petermann‐Rocha et al., 2020). Deletion of the QPRT gene resulted not only in reduced muscle mass per body weight, but also in an increase in adipose tissues in various other organs, suggesting that metabolites of the kynurenine pathway may play crucial roles in changes of body composition in late life. In addition, it is interesting to note that the changes in body composition occurred most significantly at young to middle age, which is an opposite pattern of changes of neuromuscular functions with aging. For example, there were notable increases in the weight of visceral fat, liver, spleen, and heart in the younger QPRT^−/−^ mice than in wild type mice of the same age. Most kynurenine pathway enzymes are highly expressed in liver, so that compensatory metabolic changes may mitigate QA neurotoxicity in early age in liver and other peripheral organs. Ongoing studies in our laboratory are, therefore, designed to examine the levels of several kynurenine pathway metabolites in male and female QPRT^−/−^ mice of different ages.

The present study was primarily focused on motor functions in QPRT^−/−^ mice. Given the increased jitter level, reduced CMAP, and greater degree of partial denervation in QPRT^−/−^ mice, the reduced motor function we observed is likely represent motor neuron degeneration in the spinal cord. Since neurons are subject to dying‐back axonal degeneration, that is, Wallerian‐like degeneration, under metabolic stress (Cashman & Höke, 2015), we hypothesize that genetic deletion of QPRT caused metabolic stress and facilitated dying‐back axonal degeneration of spinal motor neuron, resulting in partial denervation at the neuromuscular junction. This will, in turn, increase jitter level, followed by reduced CMAP as the dying‐back axonal degeneration progresses. At the same time, emerging evidence suggests that kynurenine directly causes toxicity to skeletal muscle, possibly associated with oxidative injury and lipid peroxidation (Kaiser et al., 2019). While we did not observe much difference in grip strength between genotypes except for males, the grip is also influenced by sensory feedback and is not a sensitive measure for motor function. In our study, the force‐frequency relationship curve showed significantly reduced peak force in QPRT^−/−^ mice. At the same time, the peak force was achieved at 40‐60 Hz range in both genotypes suggesting that contractile functions of skeletal muscles of QPRT^−/−^ mice are relatively preserved. Overall muscle weights and myofiber diameter were also comparable between the two genotypes across sex and age. Taken together, our results suggest that (1) the total number of functional motor units are reduced in old QPRT^−/−^ mice, and (2) denervation has to progress to a certain extent before skeletal muscle undergoes neurogenic atrophy. Given the increased visceral fat and reduced lean body mass in QPRT^−/−^ mice, it is also possible that QPRT^−/−^ mice develop pseudo‐hypertrophy due to increased connective tissue in the skeletal muscle, masquerading muscle atrophy. In future studies, we will compare the amount of fatty replacement and connective tissue in the skeletal muscle between the two genotypes. In addition, while fragmentation of NMJ has been widely regarded as an early sign of denervation (Pratt et al., 2013), there are also opinions that fragmentation is in fact a compensatory reaction and may suggest regeneration rather than degeneration of NMJ (Slater, 2020). It is important to note that NMJ structure is dynamic and subject to remodeling under various circumstances. For example, Valdez et al. previously showed that exercise and calorie restriction reversed age‐related changes in NMJ morphology in 24‐month‐old mice (Valdez et al., 2010). The fragmentation of NMJ in QPRT^−/−^ mice likely reflects the active remodeling of NMJ structure.

Interestingly, our fatiguability study showed that gastrocnemius muscles of QPRT^−/−^ mice are more resistant to repeated tibial nerve stimulation than muscles in young and old male wild type mice. This is likely due to fiber type I predominance, which is seen in age‐associated muscle weakness (Coggan et al., 1992; Lexell et al., 1986, 1988). This fast‐to‐slow transition with aging has been documented in numerous histological, physiological, and proteomic studies in both human and animal models (Doran et al., 2009; Einsiedel & Luff, 1992; Lexell et al., 1986; Vaillancourt et al., 2003). It appears that the fast‐to‐slow transition of muscle fiber type was accelerated in male QPRT^−/−^ mice. As shown in Figure 4b, the fatigability trajectory of male QPRT^−/−^ mice is slightly, but significantly, higher than that of male wild type mice, and considering that resistance to fatigue is the key feature of type I myofibers, our results may suggest type I predominance in male QPRT^−/−^ mice. This also may explain why the initial isometric force of wild type mice is slightly higher than that of QPRT^−/−^ mice, despite the fact that the maximal isometric force of wild type mice is significantly higher than that of QPRT^−/−^ mice in old males (Figure 4b). We performed the fatigability protocol a few minutes after the force‐frequency protocol where mice were stimulated 8 times before the fatigability test. Although we gave about 3–5 min between the two protocols, muscle fatigue from the previous protocol was unavoidable. Since muscles of wild type mice fatigue faster than those of QPRT^−/−^ mice, the isometric force of wild type mice at baseline was slightly lower than that of QPRT^−/−^ mice in old males when fatigability protocol started. This pattern was not observed in females because there was no difference in fatigability between old wild type and QPRT^−/−^ mice. Overall, given that fiber types are determined by motor neurons, this fiber type switch further suggests that neurogenic atrophy plays an important role in age‐associated muscle weakness.

Our study has several limitations. First, QPRT elimination in our knockout mice is not tissue specific. Therefore, we cannot tell if the facilitated neurodegeneration is due to direct toxicity to neuromuscular tissues or to secondary effects of increased QA. Our previous in vitro studies suggest that QA has direct toxicity to motor neurons (Westbrook et al., 2020), and we are planning to eliminate the QPRT gene conditionally in specific tissues, such as motor neurons and myocytes. Second, we have not measured kynurenine pathway metabolites upstream and downstream from QA in the neuromuscular tissues at various age points. This includes most notably 3‐HK, which can synergistically potentiate the toxicity of QA (Westbrook et al., 2020). Due to their low levels in neuromuscular tissues, it is technically challenging to measure various kynurenine pathway metabolites in the spinal cord. In Figure 1, we were able to show that QA levels were increased by about 3‐fold on average in the spinal cords of small groups of male and female QPRT^−/−^ mice of differing ages. There were not enough measurements to evaluate statistical significance between age groups in this study. In our next project, we plan to compare the levels of all tryptophan/kynurenine metabolites from spinal cords, muscle, and plasma between QPRT^−/−^ and wild type mice in larger groups of mice from younger‐, middle‐aged, and older‐age groups. Third, the isometric forces were normalized to body weight, not muscle weight. Given that there is a difference in the body composition between the two genotypes, it is not clear whether the differences in isometric force were mainly driven by body composition or not. This problem could have been avoided had we normalized the force to the muscle weight, not the total body weight. However, that was not feasible because we had to keep the mice alive for aging. Finally, it is difficult to evaluate interactions between the kynurenine pathway and other related metabolic pathways in this mouse model. It is certainly possible that deletion of QPRT caused compensatory changes resulting in protection against increased QA levels. Thus, we will carry out untargeted metabolomics from skeletal muscle and spinal cords to further evaluate changes in overall metabolism in QPRT^−/−^ mice. Chemical compounds are available that can manipulate each step of the kynurenine pathway (Schwarcz & Stone, 2017). In future studies, we will attempt to reverse phenotypes either pharmacologically or genetically. Once a therapeutic target is identified, human translation to prevent sarcopenia and frailty is possible.

## 4 METHODS

### 4.1 QPRT^−/−^ mice

Mutant mice (C57BL/6J/129S background) were generated by gene trapping using an OmniBank embryonic stem cell line containing an insertion within the first intron of the *Qprt* gene. A BLAST search of the OmniBank sequence database (Zambrowicz et al., 1998) identified several mouse embryonic stem (ES) cell clones predicted to contain gene trap mutations within the *Qprt* gene (accession NM_133686). ES cell clone OST337946 was selected for further characterization based on the sequence similarity of its 3′ RACE (rapid amplification of cDNA ends) tag with the second exon of the *Qprt* gene. Inverse genomic PCR was used to amplify the vector (EU676804) insertion site from this clone and to localize the insertion mutation to intron 1. Loss of *Qprt* expression was confirmed by reverse transcription (RT)‐PCR analysis using primers complementary to exons flanking the insertion. Primers used for genotyping are as follows: knockout/knockout forward (CGACACCTGGGTTCCGACTGGT) and reverse (CCTGCCGAGCCTTCAGCACTGC) 398 bp targeted primers and wild type forward (TGGGCACAACAGACAATCGG) and reverse (ACTTCGCCCAATAGCAGCCAG) 221 bp primers.

Mice were bred and housed at the Johns Hopkins University Bayview Vivarium and maintained under SPF barrier conditions in a 14‐h light/10‐h dark cycle kept at a temperature automated range between 22°C ± 3.6°C (Siemens Building Technologies Inc.). More specifically, animals were housed in high‐temperature polycarbonate 75 in^2^ autoclaved cages in ventilated racks (Allentown Inc.). Bedding consisted of autoclaved corncob from Harlan Teklad. Autoclaved chow (Harlan, Teklad 2018SX), and reverse osmosis–filtered hyperchlorinated water were always provided to animals (Edstrom Industries). Animal cages were changed every 2 weeks within animal research sterile fume hoods (The Baker Co.) disinfected with MB‐10 disinfectant spray (Quip Laboratories Inc.). Mice were monitored daily, and body and tissue weights were recorded from each mouse using a standard analytical scale (Mettler Toledo, LCC).

### 4.2 QA Measurement

Spinal cord tissues from QPRT^−/−^ and wild type mice were homogenized in 0.1% ascorbic acid (1:20, v/v) for the determination of QA. Fifty microliters of an internal standard ([^2^H_3_]quinolinic acid) were added to 50 μL of the tissue preparation, and proteins were precipitated with 50 μL of acetone. After centrifugation (13,700 *g*, 5 min), 50 μL of methanol: chloroform (20:50) were added to the supernatant, and the samples were centrifuged (13,700 *g*, 10 min). The upper layer was added to a glass tube and dried down for 90 min. The samples were then derivatized with 120 μL of 2,2,3,3,3‐pentafluoro‐1‐propanol and 130 μL of pentafluoropropionic anhydride at 75°C for 30 min, dried down and reconstituted in 50 μL of ethyl acetate. One μL was injected in the GC/MS (Notarangelo et al., 2012).

### 4.3 Anesthesia

All experimental procedures were performed under inhalation anesthesia of isoflurane (Kent Scientific SomnoSuite® Small Animal Anesthesia system). Animals were first exposed to 3%–5% anesthesia and maintained using 1%–2% anesthesia with a flow rate of 0.5–1 L/min. A far Infrared Warming Pad was used for body heat support during the procedure to reduce the risk for hypothermia. Animals were monitored throughout the entire procedure to ensure a normal undisturbed heart (310–840 bpm) and respiratory rate (80–230 bpm).

### 4.4 Electrophysiology

Adult QPRT^−/−^ and wild type mice at different age groups were subject to electrophysiological studies using Cadwell® Sierra Summit™ EMG/NCV/EP system, disposable electrodes (0.5″27G), and disposable monopolar needles (75 mm × 26G). The same measurement of CMAP and single fiber action potential in mice has been described in previous publications (Chung et al., 2017, 2018; Iyer et al., 2021; Sheth et al., 2018). In short, compound motor action potential (CMAP) measurements began by anesthetizing animals and placing them in a prone position. A transcutaneous reference needle was placed just medial of the sciatic notch close to the erector spinae muscles toward the tibial nerve branch, and a stimulating needle was placed on the notch itself. Another recording needle was placed within the gastrocnemius muscle and a reference electrode in the adjacent tendons. A transcutaneous ground electrode was placed near the base of the tail. Stimulatory intensity (mA) was gradually increased to optimize O‐P amplitude (mV) for compound motor action potential (CMAP). The maximal values of CMAP amplitudes were taken when the amplitudes were no longer increased at supramaximal stimulation. For jitter measurement, a reusable, 27‐gauge, 25‐mm single fiber needle (Technomed Europe, Maastricht‐Airport, Netherlands) was inserted into the gastrocnemius muscle. A stimulating and its reference needles were inserted in the same places as in the CMAP setting. Motor axons were stimulated at a rate of 10 Hz. The filter was set at 500 Hz to 10 kHz, and the gain ranged from 100 μV to 1 mV. A single fiber action potential was defined by a potential that arose within 2 ms from stimulation potential and was greater than 200 μV in amplitude with rise time of less than 200 μs. One hundred stimulatory potentials were collected calculate jitter in at least 10 different stimulation potentials per animal.

### 4.5 Muscle isometric force measurements

Young, middle‐aged, and old mice were utilized for nerve‐evoked isometric force evaluation of gastrocnemius muscles as previously described (Olojo et al., 2011; Ward et al., 2018; Westbrook et al., 2020). The overall assessment consisted of anesthetizing the mice and placing them in a supine position with the knee position fixed and foot secured to the footplate (300C‐FP) of an Aurora Scientific© Incorporated High‐Power Bi‐Phase Current Stimulator (701B) and Dual‐Mode Lever System (305C‐LR‐FP). Disposable Subdermal Needle Electrodes (0.5″27G) were placed both in the tibial branch of the sciatic nerve (stimulatory) and adjacent muscle tendons (reference) to stimulate the gastrocnemius muscle. Electrical current was individually adjusted to produce maximal isometric force before isometric force testing. The force versus frequency relationship was determined with 500 μs trains of pulses between 1 and 150 Hz. In addition, the fatigability of the gastrocnemius muscle was assessed immediately after force‐frequency assessment. With the mouse and electrical probes in the same position, the nerve was stimulated every 5000 μs for 500 μs at 60 Hz for a total of 10 min. DMAv5.501 analysis software (Aurora Scientific) was used to analyze data.

### 4.6 Frailty index

A simple, noninvasive total quantification of the frailty of each age group was assessed and compared using the exact methods and assessment forms developed by Whitehead et al. (2014). A total of 31 clinical signs of aging were observed to evaluate the neuromuscular, sensory, respiratory, digestive, and urogenital systems. Signs of physical discomfort, body weight (g), and temperature (Goodbaby Dual‐Mode Thermometer) were also collected and scored based on deviations from the mean score established from young animals. Scoring of each frailty item was performed as follows: 0 indicated no sign of deficit, 0.5 eluted mild deficit, and 1 indicated severe deficit. Examinations were completed at approximately the same time for each group. Additional materials included a dog training clicker used to evaluate the auditory system and a paintbrush used to evaluate the mouse grimace scale.

### 4.7 Immunofluorescent staining and quantification of neuromuscular junction (NMJ)

Extensor digitorum longus (EDL) muscles from age groups mentioned above were dissected, fixed, and immunologically stained as previously described (Chung et al., 2017; Pratt et al., 2017; Westbrook et al., 2020). In short, muscles were fixed for 20 min in 4% paraformaldehyde 1× PBS (pH 7.4). Muscles were then placed in 15% sucrose 1× PBS (overnight at 4°C), followed by 30% sucrose 1× PBS for a minimum of 24 h. Muscle tissues were embedded in cryostat OCT embedding media and cut into longitudinal 40 μm sections, which were then placed in a 24‐well plate filled with 1× PBS. Before immunofluorescent staining, tissues were permeabilized with 0.4% Triton and 1× PBS at room temperature for 1 h and then blocked with 5% normal horse serum, 5% Tween 20, and 1× PBS blocking buffer for 1 h at room temperature. For the presynaptic marker, sections were labeled with anti‐*β*III‐tubulin mAb (1:1000; Promega, catalog G7121) antibody for 48 h at 4°C, followed by secondary fluorescein isothiocyanate–labeled horse anti‐mouse (1:200; Vector Laboratories, catalog FI‐2000) and α‐bungarotoxin (1:1000, Invitrogen, catalog B13423) for a postsynaptic marker. Stained sections were examined using z‐stack 40X objective confocal microscopy (Zeiss LSM 880). At least 20 different images, containing ~50–100 NMJs, were obtained per animal. The laser confocal images of NMJ were reconstructed to 3D images. 3D volumes of presynaptic and postsynaptic junction were measured using Imaris 9.5 F1 software, and the volumetric ratio of presynaptic over postsynaptic junction was calculated by two individuals (TC and TB) in a blinded way.

### 4.8 Muscle histology

Subset of five wild type and five QPRT^−/−^ mice per age group were euthanized and perfused with PBS before muscle extraction. Immediately after tissue collection, samples were weighed, snap‐frozen in dry‐ice‐cooled isopentane and stored at −80°C for subsequent morphological analysis. Staining procedures were followed as previously described (Ward et al., 2018). Cryosections were cut from the middle of the gastrocnemius and tibialis anterior muscles, air‐dried to a glass microscope slide, fixed with 4% paraformaldehyde, and labeled with Alexa‐488–conjugated wheat germ agglutinin (WGA; 1 mg/mL at 1:5000 in 1× PBS, Thermo Fisher Scientific W11261) to illuminate each myofibril. Muscle sections were imaged under 20× objective, high‐resolution wide‐field introverted GFP fluorescence BZ‐X Series Keyence microscope (Keyence Corporation of America). Cross‐sectional areas of each muscle fiber were measured using the Myosight plugin for ImageJ and Fiji software.

### 4.9 Statistics

Our main question was to examine the effects of QA toxicity on frailty index, body composition, and grip strength, and how the effects may vary by age and sex (i.e., effect modification). Because the mice used in the study were independently sampled from different age/sex groups, an unpaired *t*‐test was used to compare frailty index, body composition, and grip strength between the two genotypes by age and sex. For SFEMG jitter and NMJ volumetric analyses, the jitter distribution did not have normal distribution when tested with Kolmogorov–Smirnov and Shapiro–Wilk tests. Therefore, we used nonparametric Mann–Whitney test. Repeated measures ANOVA with Huynh‐Feldt corrected *p*‐value was used to test for genotype‐frequency interaction on force across the frequency spectrum of stimulation rate within each age group. To compare fatigability curves, we used mixed‐effects models with random intercept and random linear and quadratic time effects. Two‐way ANOVA analyses of CMAP, grip strength, and jitter levels were conducted to examine the group differences by age and genotype as well as their interaction. Kaplan–Meier survival analysis was employed to compare survival between the two genotypes with cox regression after adjusting for sex. A *p*‐value less than 0.05 was considered significant. Stata and GraphPad Prism software programs were used to analyze and present the data.

### 4.10 Study approval

All procedures were approved by the Animal Care and Use Committee of the Johns Hopkins University before experimentation and closely followed guidelines set forth by the Care and Use of Laboratory Animals Guide (8th ed., National Research Council (US), 2011).

## AUTHOR CONTRIBUTIONS

Tae Chung conceived and planned the idea. Tae Chung oversaw the direction and planning. Tae Chung, Taylor Bopp, Francesca M. Notarangelo, and Chris Ward carried out experiments. Robert Schwarcz, Jeremy Walston, and Ahmet Hoke supervised the experiments. Qian‐Li Xue provided statistical support. Reyhan Westbrook assisted with data interpretation and reviewed the manuscript. All authors provided critical feedback and helped shape the research, analysis, and manuscript.

## CONFLICT OF INTEREST STATEMENT

None.

## Supporting information


Figure S1

Figure S2

Table S1

## Data Availability

The data that support the findings of this study are available on request from the corresponding author Tae Chung. The data are not publicly available due to privacy or ethical restrictions.
