# Supplementary figures and images for: Deletion of quinolinate phosphoribosyltransferase gene accelerates frailty phenotypes and neuromuscular decline with aging in a sex‐specific pattern
Source: Aging Cell. 2023 Apr 20;22(7):e13849. doi: 10.1111/acel.13849 (PMC10352574; doi:10.1111/acel.13849)

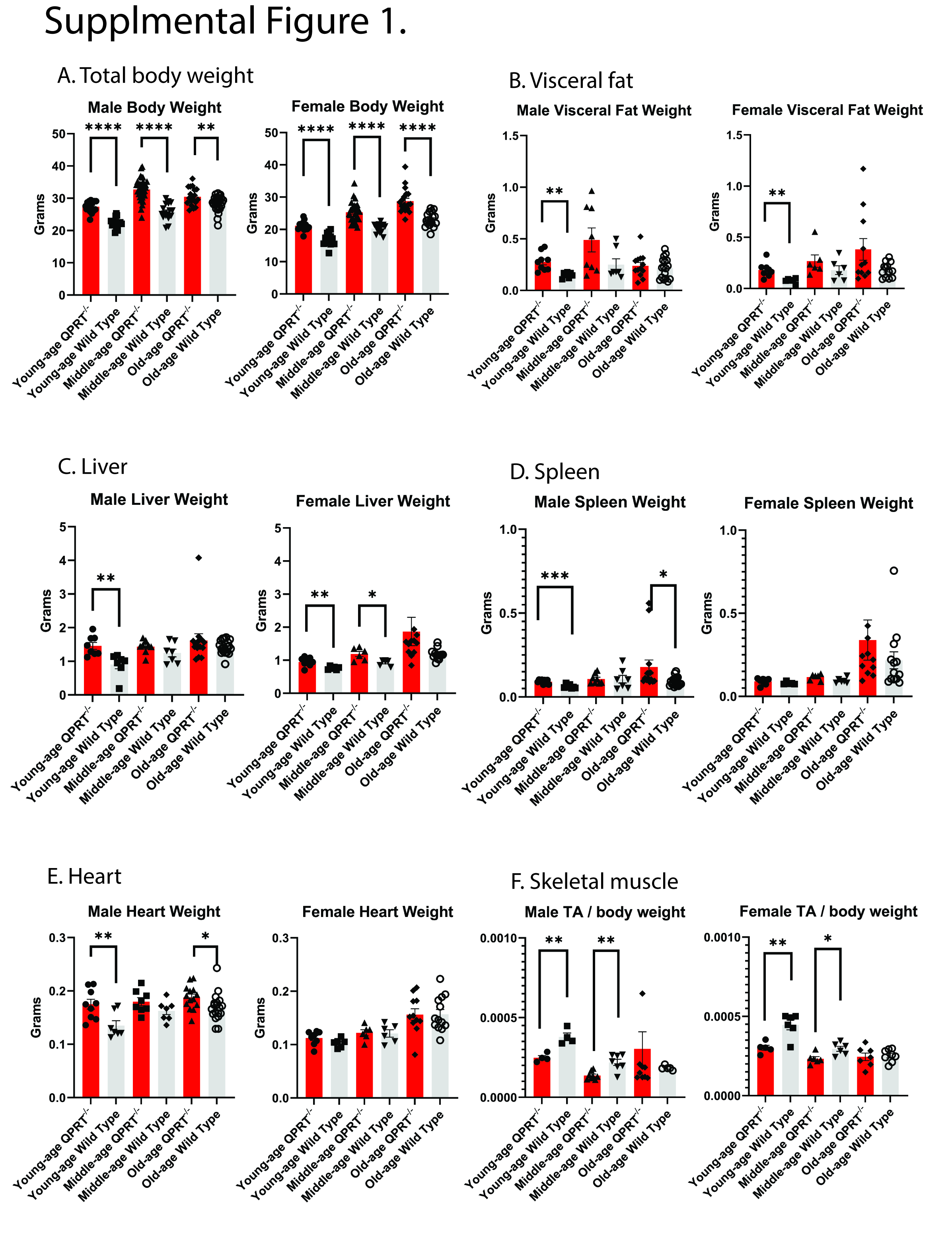

Supplement: Supplementary file 1 — Figure S1 [file ACEL-22-e13849-s001.tif]

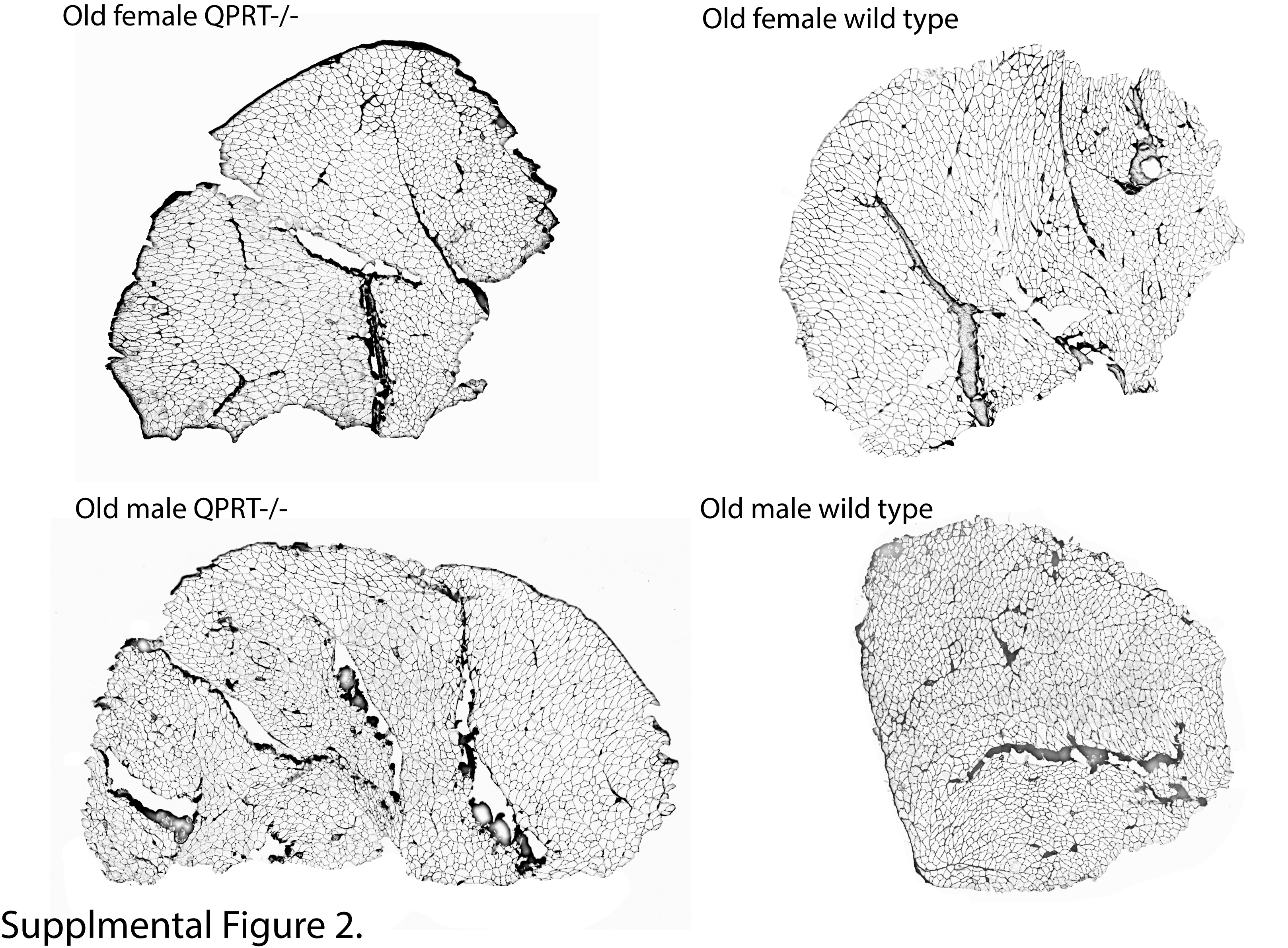

Supplement: Supplementary file 2 — Figure S2 [file ACEL-22-e13849-s002.tif]
